# Levels of organophosphate flame retardants and their metabolites among 391 volunteers in Taiwan: difference between adults and children

**DOI:** 10.3389/fpubh.2023.1186561

**Published:** 2023-08-30

**Authors:** Fu-Jen Cheng, Chih-Hwa Wang, Hsiu-Yung Pan, Chih-Cheng Chen, Wan-Ting Huang, Shau-Hsuan Li, Liang-Jen Wang, Chin-Chou Wang, Wen-Chin Lee, Kai-Fan Tsai, Yu-Che Ou, Chia-Te Kung

**Affiliations:** ^1^Department of Emergency Medicine, Kaohsiung Chang Gung Memorial Hospital, Chang Gung University College of Medicine, Kaohsiung, Taiwan; ^2^Section of Neonatology, Pediatrics Department, Kaohsiung Chang Gung Memorial Hospital, Chang Gung University College of Medicine, Kaohsiung, Taiwan; ^3^Department of Laboratory Medicine, Kaohsiung Chang Gung Memorial Hospital, Chang Gung University College of Medicine, Kaohsiung, Taiwan; ^4^Division of Hematology-Oncology, Department of Internal Medicine, Kaohsiung Chang Gung Memorial Hospital, Chang Gung University College of Medicine, Kaohsiung, Taiwan; ^5^Department of Child and Adolescent Psychiatry, Kaohsiung Chang Gung Memorial Hospital, Chang Gung University College of Medicine, Kaohsiung, Taiwan; ^6^Department of Occupational Medicine, Kaohsiung Chang Gung Memorial Hospital, Chang Gung University College of Medicine, Kaohsiung, Taiwan; ^7^Division of Nephrology, Department of Internal Medicine, Kaohsiung Chang Gung Memorial Hospital, Chang Gung University College of Medicine, Kaohsiung, Taiwan; ^8^Department of Obstetrics and Gynecology, Kaohsiung Chang Gung Memorial Hospital, Chang Gung University College of Medicine, Kaohsiung, Taiwan

**Keywords:** organophosphate flame retardant, OPFR, OPFR metabolites, Tris(2-butoxyethyl) phosphate, Taiwan

## Abstract

**Background:**

Organophosphate flame retardants (OPFRs) are ubiquitous in the environment. The compositions and concentrations of different OPFRs metabolites vary in different environments depending on different human activities. The objective of the present study was to evaluate the exposure of different age groups to OPFRs in Taiwan.

**Methods:**

Volunteers provided urine samples and responded to questionnaires including demographic factors, underlying disease, lifestyle information, and occupation from October 2021 to January 2022. OPFR measurements were performed using a Waters Acquity Ultra-Performance Liquid Chromatography system coupled with a Waters Xevo TQ-XS mass spectrometer.

**Results:**

A total of 391 volunteers (74 children and 317 adults) were enrolled in this study. The concentrations (presented as μg/g creatinine) of bis(1,3-dichloro-2-propyl) phosphate (BDCPP, *p* = 0.029) and tri-n-butyl phosphate (TNBP, *p* = 0.008) were higher in the adult group, while the concentrations of bis-2-chloroethyl phosphate (BCEP, *p* = 0.024), diphenyl phosphate (DPHP, *p* < 0.001), tris(1,3-dichloro-2-propyl) phosphate (TDCPP, *p* = 0.009), and Tris(2-butoxyethyl) phosphate (TBEP, *p* = 0.007) were higher in the child group. Compared with school age children (>6 years), the concentration of di(2-n-butoxyethyl) phthalate (DBEP, 1.14 vs. 0.20 μg/g creatinine, *p* = 0.001), DPHP (1.23 vs. 0.54 μg/g creatinine, *p* = 0.036), TBEP (1.63 vs. 0.29 μg/g creatinine, *p* < 0.001), and the sum of OPFR metabolites (ΣOPFRs, 6.58 vs. 2.04 μg/g creatinine, *p* < 0.001) were statistically higher in preschool-aged children. After adjusting for confounding factors, pre-school age [odds ratio (OR): 4.579, 95% confidence interval (CI): 1.389–13.115] and current smoker (OR: 5.328, 95%CI: 1.858–14.955) were independently associated with the risk of ΣOPFRs higher than 90 percentile.

**Conclusion:**

This study revealed the distribution of different OPFRs metabolites in children and adults. DBEP, DPHP, TBEP, and ΣOPFR were higher in preschool-aged children. Pre-school age and current smoking status were independent risk factors for ΣOPFRs higher than 90 percentile.

## 1. Introduction

Organophosphate flame retardants (OPFRs) were introduced in the early twentieth century and became the major flame retardant in the market after polybrominated diphenyl ethers (PBDEs) were banned and phased-out. OPFRs are incorporated by physical addition, therefore, they are easily released into the environment through abrasion, leaching, wearing, and volatilization (1, 2). In fact, OPFRs can be emitted into the environment during production, transport, and application (3). OPFRs have been detected in various environments, such as air, dust, water (including lakes, rivers, oceans, drinking water, etc.), soil, and sediments, as well as in human samples and biota (4–7). Adverse health effects have been reported with some OPFRs. For instance, tris (2-carboxylethyl) phosphine (TCEP) has been listed as a carcinogen by the California Environmental Protection Agency since 1992 (8) and classified as a Category 2 carcinogen by the European Union (9). Tris(2-butoxyethyl) phosphate (TBEP) has been shown to induce developmental toxicity in zebrafish (10) and abnormal sperm morphology and testicular pathology in rats (11). Tri-n-butyl phosphate (TNBP) and triphenyl phosphate (TPHP) have been reported to be neurotoxic to zebrafish larvae and rats (12, 13). The dominant compounds of the OPFRs detected in different environmental media varied between different regions and might be influenced by human activity. For example, tris (1-chloro-2-propyl) phosphate (TCPP) (Australia, France, Germany, UK, USA), TBEP (Austria, Italy, Spain), and TCEP (China) are the three dominant compounds found in surface water. The concentrations of organophosphate esters (OPEs), one type of OPFRs, in dust are higher in more industrialized countries (such as the US, Canada, Norway, Sweden, the Netherlands, South Korea, and Australia) than in less industrialized countries (such as Colombia, Romania, Pakistan, Saudi Arabia, Kuwait, Nepal, Philippines, India, and Vietnam). Humans are exposed to OPFRs via various pathways, including air inhalation, dust ingestion, diet ingestion, and dermal contact with diet ingestion, which are reported to be the most significant pathways (14). In one study, the median estimated daily intakes (EDI) of OPEs through dust ingestion were around 0.29–64.8 ng/kg body weight (bw)/day for children and 0.07–14.9 ng/kg bw/day for adults (15).

The difference between adults and children might be related to differences in the major pathways of exposure. Human exposure to OPFRs has been confirmed by the frequent detection of OPFR metabolites in urine. For examples, bis(1-chloro-2-propyl) 1-hydroxy-2-propyl phosphate (BCIPHIPP) and diphenyl phosphate (DPHP) were reported to be the two most abundant compounds in urine in one study where they accounted for 46 and 39% of the total amount of OPFRs (16). The highest level of DPHP was found in Australia, with geometric means (GM) of 24,400 pg./mL and 63,500 pg./mL from the two sample groups, respectively (17). High level of DPHP (GM = 9,000 pg./mL) was observed in the urine samples collected from US gymnasts after training. Among three different studies of populations in North Carolina, a similar range of bis(1,3-dichloro-2-propyl) phosphate (BDCPP) was found, with GMs of 1,800 pg./mL in pregnant women (18), 2,300 pg./mL in infants (19), and 2,320 pg./mL in the general population (20). 2-ethylhexyl diphenyl phosphate (EHDPHP), tris(2-chloropropyl) phosphate (TCPP), and TBEP were reported to be the three major OPFRs that participants had been exposed to in other studies (6, 7). The varied urine levels and compositions of OPFRs probably imply differences in exposure sources, pathways, and intensities among different countries and populations. Currently, only limited data regarding human exposure to OPFRs in Taiwan is available (21). It is also unclear whether there is a difference in the exposure to OPFRs between adults and children. Our study thus aims to analyze urine samples from volunteers to identify the composition of OPFRs in human bodies of people in Taiwan and to compare the difference in exposure and composition between adults and children.

## 2. Materials and methods

### 2.1. Study population

From October 2021 to January 2022, 391 volunteers were recruited to investigate OPFR metabolites in their urine samples. The volunteers were relatively healthy without a history of major comorbidities, such as renal disease, stroke, malignancy, or myocardial infarction. After agreeing to the study design, each volunteer provided written informed consent and completed a face-to-face questionnaire. Demographic factors, such as age, sex, and underlying diseases, and lifestyle information, such as alcohol consumption, smoking, and occupation, were collected through the questionnaire. Urine samples were collected in polypropylene tubes, sub-aliquoted into 1.5 mL tubes, and then stored at −80°C until extraction.

### 2.2. Determination of OPFR metabolites and urine creatinine

OPFR measurements were performed using a Waters Acquity Ultra Performance Liquid Chromatography (UPLC) system (Milford) coupled with a Waters Xevo TQ-XS mass spectrometer (Milford), operated in either negative or positive electrospray ionization (ESI) mode. The methods of OPFRs analysis have been described in a previous study (21). Briefly, all OPFRs and metabolites were separated using a Waters Acquity UPLC BEH Phenyl column preceded by a Waters XBridge BEH C18 Direct Connect HP-isolated column. The mobile phases were 0.5% formic acid in water (A) and 0.5% formic acid in methanol (B). The gradient was increased from initial 5 to 50% of solvent (B) linearly within 0.75 min. Then the mobile phase (B) was increased to 100% in another 3 min and held for 4.5 min. Finally, the gradient was decreased to the initial 5% of solvent (B) for a 2 min re-equilibrium. The target analytes were identified according to the retention time and ratio of the two selected precursor-ion-produced ion transitions compared with those of the standards. The concentrations of OPFRs were quantified using 12-point calibration curves (ranged from 0.02 ppb to 50 ppb with a two-fold dilution), the limits of quantitation (LOQs) were 0.02 ng/mL (TNBP, DNBP, TBEP, DEBP, TPHP, DPHP, TDCPP, BDCPP, TCEP) and 0.05 ng/mL (BCEP) and the limit of detection (LOD) was shown in Supplementary Table S1. Their recoveries were performed using the internal standard method based on individual isotope-labeled internal standards were also shown in Supplementary Table S1.

Urine creatinine was measured by colorimetric Jaffe method using the MeDiPRO creatinine kinase test (Formosa Biomedical Technology, Taipei, Taiwan). The assay was performed using an automatic biochemical analyzer Hitachi LABOSPECT 008 (Hitachi, Yokohama, Japan). Creatinine standard solution was purchased from Wako Pure Chemical Corporation (Osaka, Japan). Urine quality control material was purchased from Bio-Rad (Hercules, California, United States). The method was standardized according to the isotope dilution mass spectrometry (IDMS) traceable National Institute of Standards and Technology Standard Reference Material (NIST SRM) 967. This study was approved by the Institutional Review Board of Chang Gung Memorial Hospital (IRB no:202001031A3 and NO:202001028A3) and was conducted in accordance with the Code of Ethics of the World Medical Association (Declaration of Helsinki).

### 2.3. Statistical analysis

The OPFR metabolite concentrations were presented in two ways: micrograms per liter [μg/L] and micrograms per gram of creatinine [μg/g creatinine]. Normally distributed continuous data are presented as mean ± standard deviation (SD), and non-normally distributed continuous data are presented as the mean with 95% confidence intervals (CIs). The independent *t*-test and Mann–Whitney U test were used to examine the differences in the distribution of continuous variables. Chi-square tests were used to assess the differences in categorical variables. Binary logistic regression model was used to examine the likelihood of having concentrations above the 90 percentile for major OPFR metabolites and calculate odds ratios (ORs), 95% CIs, and *p*-values. Statistical significance was set at *p* < 0.05. All statistical analyses were performed using SPSS version 25.0 (IBM Corp., Armonk, NY, United States).

## 3. Results

### 3.1. Demographic factors of volunteers

Table 1 shows the demographic characteristics of the 391 participants. The mean age of the study participants was 35.7 years. 192 (49.1%) volunteers were male, 35 (9.1%) had hypertension, 32 (8.3%) volunteers were current smokers, and 228 (59.4%) engaged in service industry.

**Table 1:** Demographic factors of 391 volunteers.

Demographic characteristics of volunteers (*n* = 391)	*n* (%)
Age (mean ± SD)	35.7 ± 16.7
Male	192 (49.1)
Diabetes	19 (4.9)
Hypertension	35 (9.1)
Dyslipidemia	13 (3.4)
Liver disease	12 (3.1)
Current smoker	32 (8.3)
Alcohol consumption	71 (18.5)
Manufacturing	28 (7.3)
Service industry	228 (59.4)
Student	68 (17.7)
Unemployed	60 (15.6)

### 3.2. Distribution of OPFRs and its metabolites in adults and children

Table 2 shows the detection frequency of the 10 OPFR metabolites in the adults (≥17 years) and children (<17 years) groups. The detection rates of BDCPP (*p* = 0.012) and TNBP (*p* = 0.018) were higher in the adult group, while bis(2-chloroethyl) phosphate (BCEP) (*p* = 0.005), DPHP (*p* = 0.001), tris (1,3-dichloro-2-propyl) phosphate (TDCPP) (*p* = 0.016), and TBEP (*p* = 0.042) were higher in the children group.

**Table 2:** Detection frequency of the 10 OPFRs and their metabolites in adults (≥17 years) and children (<17 years) groups.

All	Number = 391 (%)		
Demographic characteristics of volunteers	Child (*n* = 74)	Adult (*n* = 317)	*p*
Age (mean ± SD)	8.4 ± 1.1	42.1 ± 11.3	<0.001
Male	40	152	0.344
**Detection frequency**			
BDCPP	11	90	0.012
BCEP	41	119	0.005
DBEP	37	134	0.228
DNBP	6	32	0.603
DPHP	71	252	0.001
TDCPP	22	55	0.016
TCEP	8	42	0.572
TBEP	50	173	0.042
TNBP	49	251	0.018
TPHP	30	108	0.294

Table 3 shows the demographic factors of volunteers and concentrations of OPFR metabolites in the adult and child groups, reported in both μg/L and μg/g creatinine. The concentrations of BDCPP (*p* = 0.016 and 0.029) and TNBP (*p* = 0.009 and 0.008, respectively) were significantly higher in the adult group. The concentrations of BCEP (*p* = 0.020 and 0.024), DPHP (*p* < 0.001 and <0.001), TDCPP (*p* = 0.016 and 0.009), and TBEP (*p* = 0.013 and 0.007) were significantly higher in the children group. The sum of OPFR metabolites (ΣOPFRs) was not significantly different between the adult and child groups (*p* = 0.175 and 0.254, respectively).

**Table 3:** Concentrations of OPFRs and their metabolites in adult and child groups.

	Child (=74)	Adult (=317)	*p*
Male	40	152	0.344
Age	8.4 ± 4.1	42.1 ± 11.3	<0.001
**OPFRs metabolites (mean, 95% CI)**			
BDCPP (μg/L)	0.28 (0.09–0.46)	0.69 (0.48–0.89)	0.016
BDCPP (μg/g creatinine)	0.30 (0.11–0.48)	1.00 (0.70–1.32)	0.029
BCEP (μg/L)	0.44 (0.30–0.58)	0.37 (0.29–0.45)	0.02
BCEP (μg/g creatinine)	0.67 (0.34–1.00)	0.64 (0.38–0.92)	0.024
DBEP (μg/L)	0.25 (0.14–0.36)	0.33 (0.17–0.49)	0.209
DBEP (μg/g creatinine)	0.45 (0.18–0.72)	0.50 (0.26–0.74)	0.119
DNBP (μg/L)	0.00 (0.00–0.00)	0.01 (0.00–0.02)	0.561
DNBP (μg/g creatinine)	0.00 (0.00–0.01)	0.02 (0.01–0.05)	0.463
DPHP (μg/L)	0.52 (0.33–0.71)	0.92 (−0.44–2.29)	<0.001
DPHP (μg/g creatinine)	0.73 (0.46–1.00)	1.31 (−0.47–3.10)	<0.001
TDCPP (μg/L)	0.03 (0.01–0.05)	0.02 (0.01–0.03)	0.016
TDCPP (μg/g creatinine)	0.04 (0.02–0.07)	0.04 (0.01–0.08)	0.009
TCEP (μg/L)	0.25 (0.08–0.73)	0.29 (0.18–0.40)	0.63
TCEP (μg/g creatinine)	0.31 (0.07–0.54)	0.46 (0.27–0.67)	0.512
TBEP (μg/L)	0.37 (0.23–0.51)	0.22 (0.17–0.27)	0.013
TBEP (μg/g creatinine)	0.65 (0.30–1.00)	0.41 (0.26–0.57)	0.007
TNBP (μg/L)	0.03 (0.02–0.04)	0.06 (0.05–0.08)	0.009
TNBP (μg/g creatinine)	0.08 (0.02–0.14)	0.10 (0.08–0.13)	0.008
TPHP (μg/L)	0.02 (0.01–0.03)	0.02 (0.01–0.02)	0.322
TPHP (μg/g creatinine)	0.03 (0.01–0.05)	0.03 (0.02–0.05)	0.274
Sum of OPFRs (μg/L)	2.19 (1.75–2.62)	2.93 (1.50–4.35)	0.175
Sum of OPFRs (μg/g creatinine)	3.27 (2.34–4.19)	4.50 (2.59–6.43)	0.254

**Figure 1:**
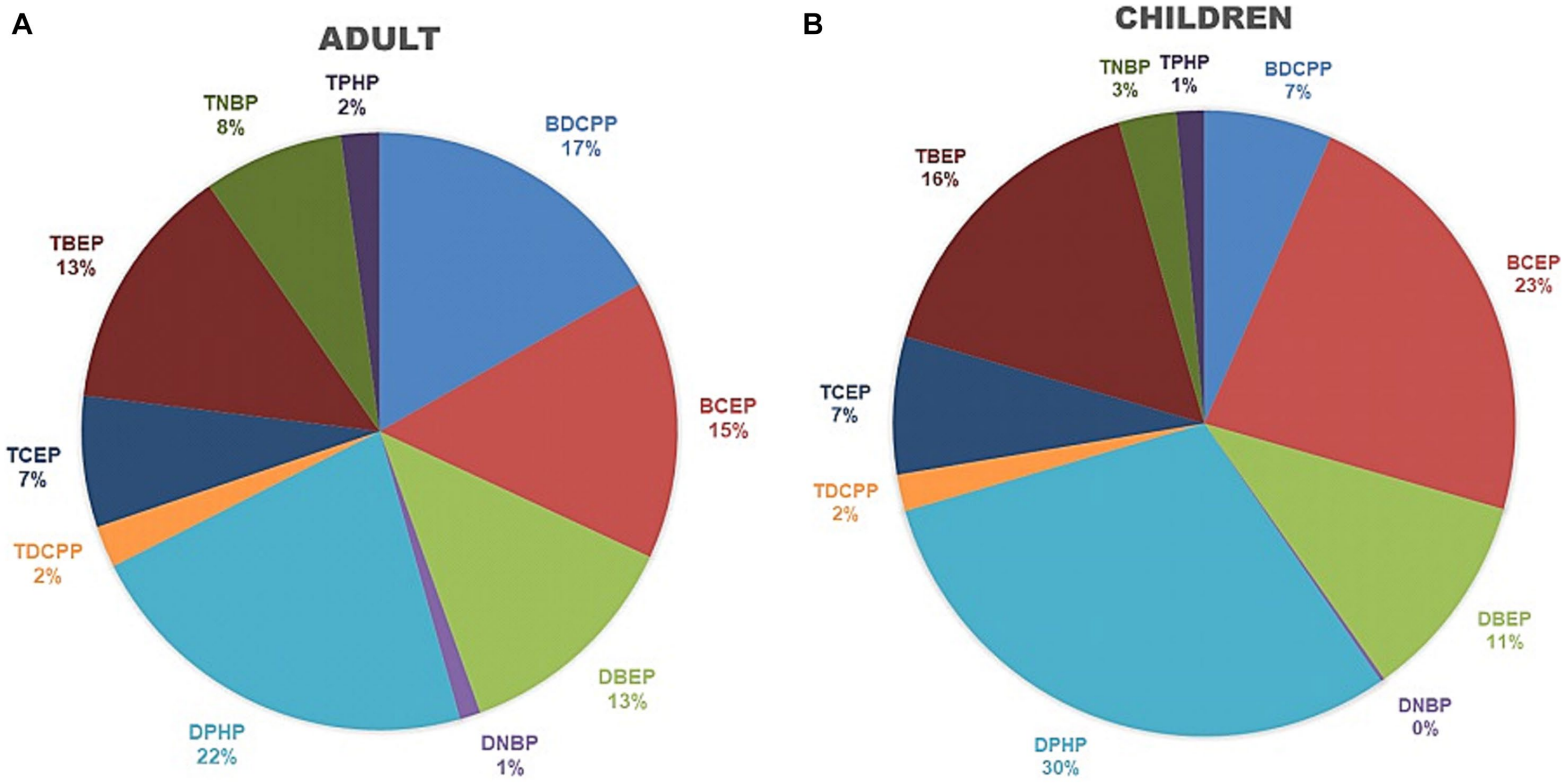
The percentage of each OPFR metabolite from the sum of OPFRs and their metabolites among adults and child groups. **(A)** Adults. **(B)** Children.

The mean percentage of each OPFR metabolite in the ΣOPFRs (μg/g creatinine) is shown in Figure 1. DPHP (21.9 ± 28.7%), BDCPP (16.8 ± 29.8%), BCEP (15.1 ± 25.1%), and TBEP (13.4 ± 20.3%) were the leading components of theΣOPFRs in adults (Figure 1A). DPHP (30.1 ± 29.9%), BCEP (22.8 ± 25.8%), TBEP (16.1 ± 2.3%), and di(2-n-butoxyethyl) phthalate (DBEP) (10.8 ± 14.9) were the leading components of the ΣOPFRs in children (Figure 1B).

### 3.3. OPFR metabolites distribution among different age groups

Table 4 shows the concentrations of OPFR metabolites among preschool-aged children (≤6 years) and school-aged children (>6 years), represented in μg/L and μg/g creatinine. The concentrations of DBEP (μg/g creatinine, *p* = 0.001), DPHP (μg/g creatinine, *p* = 0.036), TDCPP (μg/g creatinine, *p* = 0.044), TBEP (*p* = 0.004 and *p* < 0.001), TNBP (*p* = 0.032 and 0.004), and ΣOPFRs (*p* = 0.043 and <0.001) were significantly higher in the preschool-aged children.

**Table 4:** Concentrations of OPFRs and their metabolites among pre-school-age children (≤6 years) and school-age children (>6 years).

Demographic characteristics of volunteers	≤6 years (*n* = 20)	>6 yeas (*n* = 54)	*p*
Age (mean ± SD)	4.8 ± 2.2	11.5 ± 2.4	<0.001
Male	18	22	
OPFRs	Mean (95% CI)	Mean (95% CI)	
BDCPP (μg/L)	0.26 (−0.01–0.54)	0.28 (0.04–0.52)	0.529
BDCPP (μg/g creatinine)	0.44 (−0.05–0.92)	0.24 (0.06–0.43)	0.443
BCEP (μg/L)	0.65 (0.26–1.03)	0.36 (0.24–0.49)	0.221
BCEP (μg/g creatinine)	1.47 (0.31–2.63)	0.38 (0.23–0.53)	0.1
DBEP (μg/L)	0.46 (0.12–0.80)	0.18 (0.09–0.26)	0.01
DBEP (μg/g creatinine)	1.14 (0.18–2.09)	0.20 (0.10–0.30)	0.001
DNBP (μg/L)	0.00 (0.00–0.00)	0.00 (0.00–0.00)	0.58
DNBP (μg/g creatinine)	0.00 (0.00–0.01)	0.00 (0.00–0.01)	0.598
DPHP (μg/L)	0.60 (0.19–1.00)	0.49 (0.27–0.71)	0.425
DPHP (μg/g creatinine)	1.23 (0.42–2.04)	0.54 (0.32–0.76)	0.036
TDCPP (μg/L)	0.04 (0.00–0.08)	0.03 (0.01–0.05)	0.089
TDCPP (μg/g creatinine)	0.08 (0.02–0.14)	0.03 (0.01–0.06)	0.044
TCEP (μg/L)	0.25 (−0.11–0.62)	0.24 (0.05–0.44)	0.937
TCEP (μg/g creatinine)	0.34 (−0.17–0.85)	0.29 (0.03–0.56)	0.91
TBEP (μg/L)	0.62 (0.23–1.02)	0.27 (0.14–0.40)	0.004
TBEP (μg/g creatinine)	1.63 (0.41–2.85)	0.29 (0.16–0.42)	<0.001
TNBP (μg/L)	0.04 (0.03–0.06)	0.03 (0.02–0.03)	0.032
TNBP (μg/g creatinine)	0.2 (−0.01–0.41)	0.03 (0.02–0.05)	0.004
TPHP (μg/L)	0.01 (0.00–0.03)	0.02 (0.01–0.04)	0.967
TPHP (μg/g creatinine)	0.05 (0.00–0.01)	0.02 (0.01–0.03)	0.612
Sum of OPFRs (μg/L)	2.94 (1.91–3.98)	1.91 (1.45–2.36)	0.043
Sum of OPFRs (μg/g creatinine)	6.58 (3.67–9.49)	2.04 (1.61–2.47)	<0.001

Table 5 shows the results of OPFR metabolites in younger adults (≤40 years) and older adults (>40 years). The difference in all OPFR metabolites between these two groups was not statistically significant. The mean level of ΣOPFRs metabolites was 2.94 μg/L (6.58 μg/g creatinine) in the younger adult group and 1.91 μg/L (2.04 μg/g creatinine) in the older adult group.

**Table 5:** OPFRs and their metabolites in younger adults (≤40 years) group and older adults (>40 years) group.

All	Number=	%	
Demographic characteristics of volunteers	≤40 years (*n* = 152)	>40 (*n* = 165)	*p*
Age (mean ± SD)	33.0 ± 5.4	50.5 ± 8.4	<0.001
Male	65	87	0.076
OPFRs	Mean (95% CI)	Mean (95% CI)	
BDCPP (μg/L)	0.53 (0.36–0.71)	0.83 (0.46–1.19)	0.509
BDCPP (μg/g creatinine)	0.89 (0.45, 1.33)	1.55 (0, 0.97)	0.478
BCEP (μg/L)	0.32 (0.22–0.42)	0.42 (0.29–0.54)	0.872
BCEP (μg/g creatinine)	0.42 (0.03, 0.80)	0.85 (0.48, 1.22)	0.907
DBEP (μg/L)	0.32 (0.11–0.54)	0.33 (0.09–0.57)	0.398
DBEP (μg/g creatinine)	0.53 (0.19, 0.87)	0.47 (0.14, 0.79)	0.406
DNBP (μg/L)	0.02 (0.00–0.03)	0.01 (0.00–0.02)	0.169
DNBP (μg/g creatinine)	0.03 (0.01, 0.06)	0.01 (0.01, 0.03)	0.167
DPHP (μg/L)	0.21 (0.14–0.27)	1.58 (−1.05–4.22)	0.337
DPHP (μg/g creatinine)	0.33 (−2.14, 2.90)	2.21 (−0.26, 4.68)	0.253
TDCPP (μg/L)	0.02 (0.01–0.04)	0.01 (0.00–0.02)	0.812
TDCPP (μg/g creatinine)	0.05 (0.01, 0.10)	0.02 (−0.02, 0.06)	0.854
TCEP (μg/L)	0.24 (0.13–0.36)	0.34 (0.16–0.52)	0.64
TCEP (μg/g creatinine)	0.33 (0.05, 0.61)	0.59 (0.32, 0.86)	0.667
TBEP (μg/L)	0.21 (0.14–0.28)	0.24 (0.16–0.31)	0.473
TBEP (μg/g creatinine)	0.41 (0.19, 0.62)	0.40 (0.20, 0.61)	0.294
TNBP (μg/L)	0.06 (0.05–0.07)	0.06 (0.04–0.07)	0.636
TNBP (μg/g creatinine)	0.11 (0.07, 0.14)	0.09 (0.06, 0.13)	0.624
TPHP (μg/L)	0.02 (0.01–0.03)	0.02 (0.01–0.02)	0.797
TPHP (μg/g creatinine)	0.03 (0.01, 0.04)	0.03 (0.01, 0.04)	0.627
Sum of OPFRs (μg/L)	1.96 (1.60–2.31)	3.82 (1.10–6.55)	0.3
Sum of OPFRs (μg/g creatinine)	3.27 (0.50, 6.04)	5.87 (3.20, 8.53)	0.253

### 3.4. Risk factors for concentrations of each OPFRs above the 90th percentile

The characteristics and comorbidities of the volunteers above and under the 90th percentile of major OPFRs, including BDCPP, BCEP, DBEP, DPHP, TBEP, and ΣOPFRs, are listed in Supplementary Tables S2–S7. A binary logistic regression model was used to analyze the independent factors associated with the risk of major OPFRs metabolites and ΣOPFRs higher than 90 percentile. As shown in Table 6, preschool age (*p* = 0.015) and current smoking status (*p* = 0.002) were independently associated with the risk of ΣOPFRs higher than 90 percentile. Being a student was an independent factor associated with lower risk of BDCPP higher than 90 percentile (OR = 0.154, 95% CI: 0.008–0.9, *p* = 0.036). Pre-school age (*p* = 0.005) and current smoking (*p* = 0.017) were independently associated with the risk of BCEP higher than 90 percentile. Pre-school age (*p* = 0.015), current smoking status (*p* = 0.047), and diabetes (*p* = 0.023) were independently associated with a risk of DBEP higher than 90 percentile. Pre-school age (*p* = 0.002) and diabetes (*p* = 0.013) were independently associated with a risk of DPHP higher than 90 percentile. Pre-school age (*p* = 0.009) was independently associated with TBEP higher than 90 percentile.

**Table 6:** Binary logistic regression of each independent factor associated with the risk of major OPFRs metabolite and ΣOPFRs higher than 90 percentile.

	Adjusted odds ratios for Σ OPFRs >90 percentile					Adjusted odds ratios for BDCPP >90 percentile			
	OR	95% CI		*p*		OR	95% CI		*p*
Pre-school age (<6 years)	4.579	1.389	13.115	0.015	Pre-school age (<6 years)	1.149	0.060	7.063	0.901
Male sex	0.747	0.326	1.643	0.471	Male sex	0.762	0.348	1.604	0.478
Current smoker	5.328	1.858	14.955	0.002	Current smoker	1.748	0.519	5.188	0.347
					Service industry	1.381	0.623	3.395	0.439
					Student	0.154	0.008	0.9	0.036
	**Adjusted odds ratios for BCEP >90 percentile**					**Adjusted odds ratios for DBEP >90 percentile**			
	OR	95% CI		*p*		OR	95% CI		*p*
Pre-school age (<6 years)	5.139	1.700	14.075	0.005	Pre-school age (<6 years)	4.549	1.377	13.057	0.015
Male sex	0.789	0.364	1.660	0.535	Male sex	0.754	0.340	1.613	0.470
Current smoker	3.791	1.293	10.502	0.017	Current smoker	3.222	1.016	9.430	0.047
					Diabetes	4.141	1.246	11.96	0.023
	**Adjusted odds ratios for DPHP >90 percentile**					**Adjusted odds ratios for TBEP >90 percentile**			
	OR	95% CI		*p*		OR	95% CI		*p*
Pre-school age (<6 years)	6.439	2.086	18.187	0.002	Pre-school age (<6 years)	4.636	1.517	12.852	0.009
Male sex	0.589	0.260	1.262	0.176	Male sex	2.046	0.967	4.446	0.061
Current smoker	2.603	0.714	8.400	0.139	Current smoker	1.552	0.475	4.361	0.442
Diabetes	4.583	1.422	13.339	0.013	Manufacturing	0.292	0.016	1.486	0.16
Hypertension	2.117	0.723	5.478	0.162					

## 4. Discussion

The present study is the first to evaluate the composition of major OPFRs and their metabolites in humans in Taiwan. We found a difference in the distribution among children and adults. BDCPP and TNBP were higher in adults, while BCEP, DPHP, TDCPP, and TBEP were higher in children. The BCEP, DBEP, DPHP, TBEP, TNBP, and ΣOPFRs were higher in preschool-aged children. Preschool age was an independent risk factor for ΣOPFRs, DPHP, BCEP, DBEP, DPHP, and TBEP more than 90 percentile. Current smoking was an independent risk factor for ΣOPFR, BCEP, and DBEP more than 90 percentile.

OPFRs were detected in the air, indoor dust, soil, sediment, water, and sludge. A study based on Global Atmospheric Passive sampling (GPAS) network revealed that TCEP, TBEP, TCPP, TDCPP, TPHP, triethyl phosphate (TEP), and TNBP were most frequently detected OPEs in outdoor air, with detected frequencies of 88.2, 84.0, 81.7, 75.7, 69.2, 53, and 40.2%, respectively (22). TCPP, TCEP, TBEP, and TPHP were four dominant OPEs, which accounted for 45, 18, 17, and 9% of the total concentrations of OPEs (Σ_18_OPEs). The total concentrations of OPRs in indoor air were higher than those found in outdoor air. For example, the level of ΣOPEs in indoor air was eight times higher than that in outdoor air (median:40.2 ng/m^3^ vs. 5.13 ng/m^3^) in Germany (23) and more than 100 times higher in Stockholm, Sweden (340 ng/m^3^ vs. 3.1 ng/m^3^) (24). The composition of OPFRs in air varies between different countries and across indoor microenvironments, possibly because of their diverse applications in building materials and consumer products (24). Overall, TCPP, tri-iso-butylphosphate (TiBP), TNBP, and TBEP are the most abundant OPEs in indoor air. TBEP, TCPP, TCEP, TDCPP, and TPHP were the five compounds most frequently discovered in home dust at high concentrations. Higher levels of OPFRs are found in more industrialized countries (such as the US, Canada, South Korea, the Netherlands, and Australia) than that in less industrialized countries (such as Pakistan, Nepal, Vietnam, etc.). TBEP was dominated in countries with high levels of ΣOPFRs such as Japan (25), Australia (26), Canada (27), and South Korea (15) with the contributions ranging from 30 to 97%. Data on the OPFRs concentrations and compositions in different environmental media in Taiwan are lacking. We speculated that TDCPP, TCEP, TPHP, and TBEP might be the main OPFRs in our surroundings, since their metabolites were most frequently identified in our study.

Human exposure to OPFS occurs through various pathways, including air inhalation, dust ingestion, dietary intake, and dermal adsorption. In a study conducted, it was determined that the primary route of exposure for heavier organic phosphate esters (OPEs) such as TBEP and TPHP was through ingestion of dust. On the other hand, volatile OPEs like TCEP (tris(2-chloroethyl) phosphate) and TCPP (tris(2-chloro-1-methylethyl) phosphate) were predominantly absorbed through inhalation of air (6). Indoor dust from 12 countries was investigated in another study, and the reported median estimated daily intake (EDIs) of OPEs through dust ingestion were in the range of 0.29–64.8 ng/Kg body weight (bw)/day for children and 0.07–14.9 ng/kg bw/day for adults (15). He et al. (28) compared the EDIs of nine OPEs from air, dust, and food to discover that TBEP (accounting from the total exposure: 57%) was mainly from indoor dust (ingestion and dermal contact), while tris(2-chloroisopropyl) phosphate (TCIPP) (77%), TCEP (84%), and TNBP (93%) were mainly from dietary intake. Additionally, for young children, several unique exposure pathways exist that could contribute to their exposure to OPFRs. These pathways include the use of infant products and a higher frequency of hand-to-mouth behavior, which may increase their susceptibility to OPFR exposure (19). The hand-to-mouth pathway, rapid ventilation rates in infants and toddlers, and use of infant products might result in increased OPFRs exposure through dermal contact, dust ingestion, and air inhalation, thus explaining why levels of TBEP, DPHP, and BCEP were higher in children, especially for young children in the present study. Due to the potential inhalation and dermal exposure through dust and air, increasing the frequency of vacuuming and handwashing may be effective in reducing children’s exposure to Organophosphate Flame Retardants (OPFRs). These measures could help minimize the risk associated with OPFR exposure in children (28).

Adults are inevitably exposed to OPFRs in daily life since OPFRs are ubiquitous in the environment. Increased exposure to OPFRs might result from special behaviors, such as tobacco use, cigarette use, and occupational exposure. Higher detection rates and higher concentrations of OPFRs metabolites were reported in the urine of firefighters, which might be related to a higher incidence of OPFRs exposure from work (29–31). Higher detection frequencies of DPHP in spot urine samples from nail salon workers have also been reported (32, 33), and the plausible reasons might be increased TPHP exposure from the workplace of nail polishing via air inhalation, dermal absorption, and dust ingestion. People who smoke cigarettes are supposed to be exposed to higher amounts of TPHP because the adjusted GM of DPHP for cigarette smokers is higher than that of non-smokers (34). Sun et al. (35) noticed that the urine concentrations of bis(1-chloro-2-propyl) phosphate (BCIPP) in smokers and ex-smokers were higher (GM 42.3 pg./mL and GM 0.9 pg./mL, respectively) than those in non-smokers [GM 19.7 pg./mL, <limit of quantification (LOQ)], suggesting that tobacco smoking might lead to altered metabolism which results in the formation of di(methylphenyl) phosphate (DMPP) and BCIPP from their corresponding parent compounds. Our study discovered that the detection rate and urine concentration of BDCPP and TNBP were significantly higher in the adult groups, and that current smoking was an independent risk factor for ΣOPFRs, BCEP, and DBEP more than 90 percentile.

The assessment of human OPFRs exposure through biomonitoring presents certain challenges due to the relatively short half-lives (ranging from hours to days) of OPFRs in biota (5). Considering the rapid metabolism of Organophosphate Flame Retardants (OPFRs), the analysis and assessment of exposure to OPFRs may be better focused on the excreted metabolites, as they provide a more appropriate target for analysis (17, 36). BDCPP and DPHP were the most frequently detected OPFRs in spot urine samples among the nine OPFRs [BDCPP, BCIPP, BCEP, DPHP, di-p-cresylphosphate (DPCP), di-o-cresylphosphate (DOCP), dibutyl phosphate (DNBP), dibenzyl phosphate (DBZP), tetrabromobenzoic acid (TBBA)] studied in the National Health and Nutrition Examination Survey (NHANES) in the United States during 2013–2014 (37). In this study, BDCPP and DPHP were found in approximately 92% of the samples, followed by BCEP, DNBP, and BCIPP, with the detection frequencies of 89, 81, and 61%, respectively. Moreover, the concentration was highest for DPHP (ranged <0.16–193 μg/L), followed by BDCPP (<0.11–169 μg/L), and BCEP (<0.08–110 μg/L) (37). Wang et al. (38) analyzed eight types of OPFRs [DPHP, BDCPP, DOCP+DPCP, bis(2-butoxyethyl) phosphate (BBOEP), BCIPHIPP, 4-hydroxyphenyl-diphenyl phosphate (4-HO-DPHP), dibutyl phosphate (DBP), and TCEP] in the urine samples collected from healthy volunteers and found that DPHP, DOCP + DPCP, and BCIPHIPP were detected in more than 90% of the spot and first morning voids. Several studies have documented high detection rates of BDCPP and DPHP in pregnant women and children (36, 39, 40).

In our study, DPHP, TBEP, and TNBP were the most frequently detected OPFRs in urine samples collected from children, with detection frequencies of 95.9, 67.6, and 66.2%, respectively. The concentration was highest for DPHP (ranged 0.33–0.71 μg/L), followed by BCEP (0.30–0.58 μg/L), and TBEP (0.23–0.51 μg/L). In the adult group, DPHP was detected in approximately 79.5% of the samples, followed by TNBP (79.2%), and TBEP (54.6%). The concentration was highest for DPHP (ranged 0.44–2.29 μg/L), followed by BDCPP (0.48–0.89 μg/L), BCEP (0.29–0.45 μg/L), and DBEP (0.17–0.49 μg/L). The detection frequencies of OPFRs metabolites in urine samples in our study were consistent with the results reported by Ospina et al. (37), with DPHP, TNBP, and bis(n-butyl) phosphate (BNBP) (the metabolite of TNBP) being the most frequently detected. In particular, the detection rate of TBEP was significantly higher in the study group. TBEP is a plasticizer and antifoaming agent used in paints and floor finishes (41). Besides, our findings revealed that hypertension and diabetes are independent factors with a DPHP (diphenyl phosphate) levels exceeding the 90th percentile. However, our results could not clarify whether these associations are attributed to the diseases themselves or the medications used.

TBEP was detected in dust from solid waste, e-waste dumpling sites, landfills, and wastewater treatment facilities (42). Previous studies from Japan revealed that TBEP was the most prevalent OPFRs in houses (43, 44). Studies utilizing zebrafish as an animal model reported that TBEP induced neurotoxicity and developmental impairments (45–47). Additionally, it was found to reduce body length, heart rate, survival rate, and hatching rate (48), as well as modify motor behavior and oxidative stress in zebrafish (49). TBEP was also shown to induce abnormal sperm morphology and testicular pathology in rats (11). The higher detection frequency of TBEP might have resulted from a higher amount of TBEP exposure from indoor air and home dust. Further work is needed to explore the concentrations of these OPFRs in different environmental media in Taiwan to elucidate the major sources of TBEP exposure.

BDCPP is unique to TDCPP and is the most appropriate metabolite to study TDCPP exposure. BCEP and DPHP were the corresponding metabolites of TCEP and TPHP, respectively. Reports has shown that TDCPP and TCEP increase apoptosis, decrease cell growth, alter morphology, and induce changes in the mRNA and protein expression levels of GAP43, tubulin, and NF-H (50). TCEP has been classified as a Category 2 carcinogen by the European Union (9) and has been listed as a carcinogen by the California Environmental Protection Agency since 1992 (8). The European Commission proposed restricting TCEP and TDCPP in toys intended for children (under 3 years) or those that can be put toys into their mouth (>3 years) (50). The application of TCEP was effectively banned after it was listed on the Annex XVI authorization by the European Union. The addition of halogenated OPFRs to children’s products, mattresses, furniture, and electronic enclosures was prohibited in 2017 by the United States Consumer Product Safety Commission (CPSC). TPHP is a potent monocyte carboxylase inhibitor in human blood (51), and it induces contact allergy (52). TPHP disrupts reproductive performance in zebrafish (53), and it interferes with the endocrine (54–56) and metabolic systems (57). TDCPP and TPHP found in dust are associated with alterations in male hormone levels and decreased sperm quality (58). Adverse health effects following OPFRs exposure have been previously reported. The importance of surveillance and close monitoring of OPFRs exposure, in addition to the regulation of OPFRs applications, should be emphasized.

## 5. Limitations

First, our study was conducted in a single region and a single hospital with recruitment through volunteered enrollment; therefore, the results might not be generalizable to other populations, especially for different regions or countries. Second, a single measurement of OPFRs and their metabolites in a spot urine sample was used; however, weekly or monthly variations might exist. Third, we only examined the OPFRs and their metabolites in urine samples and analyzed their compositions and concentrations in the surrounding environment. Exposure assessment was not performed either. The direct relationship between environmental exposure and our results could not be determined.

## 6. Conclusion

In conclusion, our study identified different OPFRs and their metabolite distributions in children and adults. We found that BDCPP and TNBP were higher in adults, while BCEP, DPHP, TDCPP, and TBEP were higher in children. The BCEP, DBEP, DPHP, TBEP, TNBP, and ΣOPFRs were higher in preschool-aged children. Preschool age was an independent risk factor for ΣOPFRs, DPHP, BCEP, DBEP, DPHP, and TBEP more than 90 percentile. Current smoking was an independent risk factor for ΣOPFR, BCEP, and DBEP more than 90 percentile. Since children are more vulnerable to OPFRs exposure, more works are needed to better elucidate the relationship from environmental exposure and health impacts. We would collect more samples from children and also from the surrounding environments in Taiwan in the future. We hope that the results of this study will motivate the government to pay closer attention to managing the environmental pollution caused by OPFRs and their metabolites.

## Data availability statement

The raw data supporting the conclusions of this article will be made available by the authors, without undue reservation.

## Ethics statement

The studies involving humans were approved by Institutional Review Board of Kaohsiung Chang Gung Memorial Hospital (IRB NO: 202001031A3 and NO: 202001028A3). The studies were conducted in accordance with the local legislation and institutional requirements. Written informed consent for participation in this study was provided by the participants’ legal guardians/next of kin.

## Author contributions

F-JC and C-TK contributed to conception and design of the study. F-JC organized the database. H-YP performed the statistical analysis. C-HW and H-YP wrote the first draft of the manuscript. All authors wrote sections of the manuscript. All authors contributed to the article and approved the submitted version.

## Funding

This study was supported in part by research grants from Kaohsiung Chang Gung Memorial Hospital (Grant number CMRPG8K1301-3). The sponsor played no role in the study design, data collection, analysis, interpretation, writing of the report, or the decision to submit the article for publication.

## Conflict of interest

The authors declare that the research was conducted in the absence of any commercial or financial relationships that could be construed as a potential conflict of interest.

## Publisher’s note

All claims expressed in this article are solely those of the authors and do not necessarily represent those of their affiliated organizations, or those of the publisher, the editors and the reviewers. Any product that may be evaluated in this article, or claim that may be made by its manufacturer, is not guaranteed or endorsed by the publisher.
