# Supplementary material for: Levels of organophosphate flame retardants and their metabolites among 391 volunteers in Taiwan: difference between adults and children
Source: Front Public Health. 2023 Aug 30;11:1186561. doi: 10.3389/fpubh.2023.1186561 (PMC10499440; doi:10.3389/fpubh.2023.1186561)
Supplement: Supplementary file 1 [file Table_1.DOCX]

Supplementary Table 1 The detection limits and coefficient of determination of each OPFRs and its metabolites

| **Compound** | **Detection (Range)** | **Coefficient of determination (R^2^)** | **Recovery Range** |
| --- | --- | --- | --- |
| TNBP | 0.02441 - 200 ng/mL | 0.99491 | 98.12~102.32% |
| DNBP | 0.02441 - 200 ng/mL | 0.99609 | 92.13~106.45% |
| TBEP | 0.02441 - 200 ng/mL | 0.99865 | 93.17~106.12% |
| DEBP | 0.02441 - 200 ng/mL | 0.99979 | 91.33~109.31% |
| TPHP | 0.02441 - 200 ng/mL | 0.99688 | 98.73~102.85% |
| DPHP | 0.02441 - 200 ng/mL | 0.99735 | 91.84~111.45% |
| TDCPP | 0.02441 - 200 ng/mL | 0.98779 | 96.32~104.93% |
| BDCPP | 0.02441 - 200 ng/mL | 0.99254 | 94.73~105.72% |
| TCEP | 0.022201 - 200 ng/mL | 0.99787 | 98.31~102.62% |
| BCEP | 0.04882 - 200 ng/mL | 0.99696 | 85.52~115.39% |

Table 2. Demographic factors, occupation, and cormobidities among different BDCPP concentration groups of volunteers

| Demographic characteristics of volunteers | ≦90 percentile (n=352) | >90 percentile (n=39) | p |
| --- | --- | --- | --- |
| Age (mean±SD) | 35.1±17.0 | 41.2±13.7 | 0.031 |
| Male | 157 | 17 | 0.468 |
| Diabetes | 15 | 4 | 0.083 |
| Hypertension | 33 | 2 | 0.412 |
| Dyslipidemia | 12 | 1 | 0.811 |
| Liver disease | 12 | 0 | 0.251 |
| Smoking | 27 | 5 | 0.228 |
| Alcohol comsuption | 63 | 8 | 0.606 |
| Manufacturing | 27 | 1 | 0.259 |
| Service industry | 200 | 28 | 0.034 |
| unemployed | 53 | 7 | 0.562 |
| Student | 67 | 1 | 0.012 |
| Pre-school age | 19 | 1 | 0.446 |

Table 3. Demographic factors, occupation, and cormobidities among different BCEP concentration groups of volunteers

| Demographic characteristics of volunteers | ≦90 percentile (n=351) | >90 percentile (n=40) | p |
| --- | --- | --- | --- |
| Age (mean±SD) | 35.7±16.6 | 35.7±18.6 | 0.559 |
| Male | 172 | 20 | 0.905 |
| Diabetes | 18 | 1 | 0.471 |
| Hypertension | 31 | 4 | 0.789 |
| Dyslipidemia | 11 | 2 | 0.523 |
| Liver disease | 12 | 0 | 0.237 |
| Smoking | 25 | 7 | 0.022 |
| Alcohol comsuption | 66 | 5 | 0.336 |
| Manufacturing | 25 | 3 | 0.919 |
| Service industry | 205 | 23 | 0.957 |
| unemployed | 52 | 8 | 0.375 |
| Student | 63 | 5 | 0.399 |
| Pre-school age | 14 | 6 | 0.003 |

Table 4. Demographic factors, occupation, and cormobidities among different DBEP concentration groups of volunteers

| Demographic characteristics of volunteers | ≦90 percentile (n=352) | >90 percentile (n=39) | p |
| --- | --- | --- | --- |
| Age (mean±SD) | 35.8±16.5 | 34.8±18.9 | 0.731 |
| Male | 173 | 19 | 0.959 |
| Diabetes | 14 | 5 | 0.011 |
| Hypertension | 31 | 4 | 0.702 |
| Dyslipidemia | 10 | 3 | 0.094 |
| Liver disease | 10 | 2 | 0.4 |
| Smoking | 26 | 6 | 0.067 |
| Alcohol comsuption | 62 | 9 | 0.336 |
| Manufacturing | 26 | 2 | 0.643 |
| Service industry | 206 | 22 | 0.991 |
| unemployed | 53 | 7 | 0.562 |
| Student | 62 | 6 | 0.803 |
| Pre-school age | 15 | 5 | 0.021 |

Table 5. Demographic factors, occupation, and cormobidities among different DPHP concentration groups of volunteers

| **Demographic characteristics of volunteers** | ≦90 percentile (n=352) | >90 percentile (n=39) | p |
| --- | --- | --- | --- |
| **Age (mean±SD)** | 35.9±15.9 | 34.1±23.0 | 0.53 |
| **Male** | 176 | 16 | 0.288 |
| Diabetes | 13 | 6 | 0.001 |
| Hypertension | 28 | 7 | 0.035 |
| Dyslipidemia | 10 | 3 | 0.104 |
| Liver disease | 11 | 1 | 0.856 |
| Smoking | 27 | 5 | 0.254 |
| Alcohol comsuption | 68 | 3 | 0.076 |
| Manufacturing | 28 | 0 | 0.069 |
| Service industry | 208 | 20 | 0.373 |
| unemployed | 53 | 7 | 0.617 |
| Student | 57 | 11 | 0.056 |
| Pre-school age | 14 | 6 | 0.022 |

Table 6. Demographic factors, occupation, and cormobidities among different TBEP concentration groups of volunteers

| Demographic characteristics of volunteers | ≦90 percentile (n=352) | >90 percentile (n=39) | p |
| --- | --- | --- | --- |
| Age (mean±SD) | 36.0±16.6 | 32.7±21.9 | 0.242 |
| Male | 168 | 24 | 0.102 |
| Diabetes | 17 | 2 | 0.89 |
| Hypertension | 31 | 4 | 0.702 |
| Dyslipidemia | 12 | 1 | 0.811 |
| Liver disease | 11 | 1 | 0.879 |
| Smoking | 27 | 5 | 0.228 |
| Alcohol comsuption | 64 | 7 | 0.944 |
| Manufacturing | 27 | 1 | 0.259 |
| Service industry | 207 | 21 | 0.733 |
| Unemployed | 53 | 7 | 0.562 |
| Student | 60 | 8 | 0.512 |
| Pre-school age | 14 | 6 | 0.002 |

Table 7. Demographic factors, occupation, and cormobidities among different ΣOPFRs concentration groups of volunteers

| All | ≦90 percentile (n=352) | >90 percentile (n=39) |  |
| --- | --- | --- | --- |
| Demographic characteristics of volunteers | ≦90 percentile (n=352) | >90 percentile (n=39) | p |
| Age (mean±SD) | 35.4 ± 16.6 | 38.8 ± 17.9 | 0.23 |
| Male | 173 | 19 | 0.959 |
| Child | 69 | 5 | 0.305 |
| Diabetes | 17 | 2 | 0.857 |
| Hypertension | 32 | 3 | 0.868 |
| Dyslipidemia | 12 | 1 | 0.835 |
| Liver disease | 12 | 0 | 0.258 |
| Current Smoker | 24 | 8 | 0.002 |
| Alcohol comsuption | 64 | 7 | 0.877 |
| Manufacturing | 26 | 2 | 0.674 |
| Service industry | 205 | 23 | 0.562 |
| Unemployed | 52 | 8 | 0.252 |
| Student | 15 | 5 | 0.021 |
| Pre-school age | 35.4 ± 16.6 | 38.8 ± 17.9 | 0.23 |
